# Association of plasma high-density lipoprotein cholesterol level with risk of incident dementia: a cohort study of healthy older adults

**DOI:** 10.1016/j.lanwpc.2023.100963

**Published:** 2023-11-29

**Authors:** Sultana Monira Hussain, Catherine Robb, Andrew M. Tonkin, Paul Lacaze, Trevor T.-J. Chong, Lawrence J. Beilin, Chenglong Yu, Gerald F. Watts, Joanne Ryan, Michael E. Ernst, Zhen Zhou, Johannes T. Neumann, John J. McNeil

**Affiliations:** a School of Public Health and Preventive Medicine, Monash University, Victoria, 3004, Australia; b Department of Medical Education, Melbourne Medical School, The University of Melbourne, Victoria, 3010, Australia; c Turner Institute for Brain and Mental Health, School of Psychological Sciences, Monash University, Melbourne, VIC, 3800, Australia; d Department of Neurology, Alfred Health, Melbourne, VIC, 3181, Australia; e Department of Clinical Neurosciences, St Vincent's Hospital, Melbourne, VIC, 3065, Australia; f School of Medicine, University of Western Australia, Perth, Australia; g Department of Pharmacy Practice and Science, College of Pharmacy, The University of Iowa, Iowa City, IA, USA; h Department of Cardiology, University Heart & Vascular Center Hamburg, Germany; i German Center for Cardiovascular Research (DZHK), Partner Site Hamburg/Kiel/Lübeck, Hamburg, Germany; j Department of Family Medicine, Carver College of Medicine, The University of Iowa, Iowa City, IA, USA

**Keywords:** High-density lipoprotein cholesterol, Dementia, Older adults

## Abstract

**Background:**

Recent studies have reported associations between high plasma high-density lipoprotein cholesterol (HDL-C) levels and risk of all-cause mortality, age-related macular degeneration, sepsis and fractures, but associations with dementia risk remain unclear. To determine whether high plasma HDL-C levels are associated with increased incident dementia risk in initially-healthy older people.

**Methods:**

We conducted a post-hoc analysis of the Aspirin in Reducing Events in the Elderly (ASPREE) trial; a double-blind, randomized, placebo-controlled trial of daily low-dose aspirin in healthy older people. ASPREE recruited 16,703 participants aged ≥70 years (from Australia) and 2411 participants aged ≥65 years (from the US) between 2010 and 2014. Participants had no diagnosed cardiovascular disease, dementia, physical disability, or life-threatening illness at enrolment and were cognitively healthy (3MS score ≥78). All-cause dementia was a primary trial endpoint, and determined by DSM-IV criteria. Cox regression was used to examine hazard ratios between HDL-C categories <40 mg/dL, 40–60 mg/dL (reference category), 60–80 mg/dL, and >80 mg/dL and dementia. Restricted cubic spline curves were used to determine nonlinear associations. Data analysis was performed from October 2022 to January 2023.

**Findings:**

Of the 18,668 participants, 850 (4.6%) cases of incident dementia were recorded over 6.3 (SD 1.8) years. Participants with high HDL-C (>80 mg/dL) had a 27% higher risk of dementia (HR 1.27, 95% CI 1.03, 1.58). Age stratified analyses demonstrated that the risk of incident dementia was higher in participants ≥75 years compared to participants <75 years (HR 1.42, 95% CI 1.10, 1.83 vs HR 1.02, 95% CI 0.68, 1.51). Associations remained significant after adjusting for covariates including age, sex, country of enrolment, daily exercise, education, alcohol consumption, weight change over time, non-HDL-C, HDL-C-PRS, and *APOE* genotype.

**Interpretation:**

In a population of initially-healthy older adults aged ≥75 years, high HDL-C levels were associated with increased risk of all-cause dementia.

**Funding:**

10.13039/100000002National Institutes of Health, USA; 10.13039/501100000925National Health and Medical Research Council Australia; 10.13039/501100001779Monash University (Melbourne, VIC, Australia); and the 10.13039/100008018Victorian Cancer Agency (Australia).

**Research in context**

**Evidence before this study**

While several longitudinal studies have shown an association between high high-density lipoprotein cholesterol (HDL-C) and adverse health outcomes, the evidence regarding dementia remains uncertain. A search of MEDLINE and Embase, on May 21, 2023, identified English language studies that examined elevated high-density lipoprotein cholesterol and dementia risk. Search terms, included “elevated high-density lipoprotein cholesterol”, “high high-density lipoprotein cholesterol”, “high HDL”, “elevated HDL”, “dementia”, “Alzheimer's”, “vascular” “cognitive decline”. Only one study of cohorts from Denmark was identified which suggested that high HDL-C is associated with dementia in people aged 47–68 years. Since, early onset dementia may have different pathophysiology than late onset dementia it is important to extend these results in well-characterised prospective studies of older people who are cognitively intact at the study onset.

**Added value of this study**

This is the most comprehensive study to report high HDL-C and the risk of dementia in older people. Data from the Aspirin in Reducing Events in the Elderly (ASPREE) trial, with participants free from evident cardiovascular disease, physical disability, or a chronic illness expected to limit survival to less than five years and cognitively intact, was analysed. Findings showed that high HDL-C was associated with dementia risk and the risk increased with age.

**Implications of all the available evidence**

High HDL-C is associated with an increased risk of all-cause dementia in both middle-aged and older individuals. The association appears strongest in those 75 years and above.

## Introduction

The association between an high high-density lipoprotein cholesterol (HDL-C) and a lower risk of cardiovascular disease (CVD) has been well established. However, a series of recent reports has highlighted a relationship between high HDL-C and a range of adverse health conditions.^1^, ^2^, ^3^, ^4^, ^5^ These include a recent report from two large community-based cohorts from Denmark which found an association between very high HDL-C levels and an increased future risk of all-cause dementia and Alzheimer's disease.^6^ The populations involved were middle-aged men and women whose ages ranged from 47 to 68 years.^6^ The question addressed by our study was whether high HDL-C is associated with a future increase in dementia risk in a large population of older individuals with initially intact cognitive function.

HDL-C particles are complexes of lipids and apolipoproteins, which are synthesised both in the systemic circulation and in the central nervous system. Within the central nervous system, the delivery of lipids to neurons by high-density lipoprotein (HDL) supports the synthesis of new membranes and may play a significant role in restoring synaptic connections in neurodegenerative conditions.^7^ However, at very high plasma levels, the structural components and the actions of HDL-C change, and they may become deleterious to health. For example, recent studies have identified associations between very high HDL-C levels and all-cause mortality,^1^^,^^2^ age-related macular degeneration,^3^ sepsis^4^ and fractures.^5^

The Aspirin in Reducing Events in the Elderly (ASPREE) trial was a randomised controlled trial of low-dose aspirin involving healthy Australian and US participants aged 70 and above (>65 for US minorities).^8^, ^9^, ^10^ Plasma HDL-C levels were measured at the time of enrolment. Longitudinal follow-up involved detailed measures of cognitive function, allowing assessment of dementia according to DSM-IV criteria. This provides an ideal setting to investigate the relationship between plasma HDL-C levels and incident dementia risk.

## Methods

### Study participants

This study included data from the ASPREE trial and its ongoing longitudinal follow-up study, ASPREE-XT (ASPREE—eXTension). ASPREE participants were recruited between March 2010 and December 2014 through their general practitioners in Australia and through academic health centres in the US. They were community-dwelling older adults from Australia (n = 16,703 aged ≥70 years) and the US (n = 2411, ≥65 years) with no previous diagnosis of CVD events, and physical disability, or chronic illness expected to limit survival to <5 years.^8^^,^^10^^,^^11^ Additionally, participants with a self-report or physician diagnosis of dementia at recruitment, or with a Modified Mini-Mental State Examination (3MS) score of less than 78, were also excluded.^12^ Participants were randomised to receive aspirin 100 mg daily or placebo.^9^ Ethics approval was obtained from Institutional Ethics Review Committees in Australia and the US and participants provided written informed consent. This study followed the Strengthening the Reporting of Observational Studies in Epidemiology (STROBE) reporting guidelines.

### Routine assessment

During annual in-person visits, anthropometric and laboratory measurements were recorded. Data regarding medical comorbidities, lifestyle and socio-demographic factors, prescription medications, and other related health variables were collected concurrently.^11^ Data collection was undertaken according to standard operating procedures.

### Measurement of serum cholesterol

Fasting lipids, including HDL-C and non-HDL-C, were measured by a local pathology provider from fresh plasma^10^^,^^11^ close to the time of recruitment.

### APOE genotyping and polygenic risk score for HDL-C

Peripheral blood samples were provided by ∼75% of ASPREE participants at the time of study enrolment as part of the ASPREE Healthy Ageing Biobank. Blood samples were processed to buffy coat within 4 h of collection and then stored at −80 °C. DNA was later purified from the buffy coat via magnetic bead extraction.^13^ 13,503 samples were genotyped using the Axiom 2.0 Precision Medicine Diversity Research Array (Thermo Fisher Scientific, CA). Genotyping data was quality controlled, processed and analysed following standard protocols. *APOE* genotype was directly measured at two single-nucleotide polymorphisms (SNPs), rs7412 and rs429358, which were included in the Axiom array.

In ASPREE participants, the polygenic risk score (PRS) was generated using 309 independent genome-wide significant variants (P < 5 × 10−8) associated with HDL-C. These genomic variants were yielded from a large-scale genome-wide association study (GWAS)^14^ based on ∼300,000 multi-ethnic participants in the Million Veteran Program and performed using GCTA-COJO software.^15^ The PRS was calculated by summing all these variants weighted by reported beta coefficients in the GWAS, and then converted to Z scores.^16^

### Assessment of incident dementia

Participants were assessed with the Modified Mini-Mental State (3MS) test at baseline and Year 1, and then biennially over the follow-up period. They were considered as having ‘triggered’ for further cognitive assessment if their 3MS score fell below 78 or dropped by more than 10 points from their predicted score based on their own baseline and a subsequent annual visit.^12^ Triggers could also include a report of memory or cognitive problems noted in participants' medical records, a clinician diagnosis of dementia or prescription of a cholinesterase inhibitor.^12^

After a trigger was reported additional information was collected. This included additional cognitive tests, functional evaluations and collection of reports of clinical investigation and imaging results from those where further clinical investigation had been undertaken.^12^ An adjudication panel of physicians with expertise in dementia reviewed this information confirmed a diagnosis of dementia if DSM-IV criteria were met, i.e. there was evidence of impaired memory accompanied by the presence of aphasia, apraxia, agnosia, or disturbances in executive functioning (and the cognitive impairments were severe enough to affect social and/or occupational functioning).^12^ The date of dementia onset was considered to be the date of confirmation by the adjudication committee.^12^

### Statistical analysis

For continuous variables analysis of variance (ANOVA) and for categorical variables chi-squared test were used where appropriate to compare baseline characteristics between participants who did and did not develop dementia during the follow-up period. Continuous variables were presented as mean (±SD), categorical variables were reported as frequency (proportion).

Cox regression was used to calculate the hazard ratios (HR) and 95% confidence interval (CI) between four categories of HDL-C at baseline (<40 mg/dL, 40–60 mg/dL, 60–80 mg/dL, >80 mg/dL), and incident dementia occurring after randomisation. HDL-C 40–60 mg/dL was used as the reference category. Analytical models included adjustments for one or more of age, sex, country of enrolment, physical activity (walking outside >30 min), alcohol use (never/former vs current), frailty status (determined via modified Fried frailty phenotype, which defines frailty as the presence of weakness, slowness, exhaustion, low physical activity, and weight loss),^17^ education, smoking status, aspirin, baseline cognitive abilities (3MS score), hypertension, diabetes, chronic kidney disease (eGFR <60 ml/min), non-HDL-C, *APOE* genotype, and HDL-C-PRS and 10 principal components (PC) for population structure. Stepwise regression was employed to assess the significance of baseline covariates of interest, with exclusion occurring for those with P-values ≥0.2. The associations between HDL-C and incident dementia were investigated using restricted cubic spline curves to determine whether there was evidence of a nonlinear association, treating an HDL-C level of 55 mg/dL (median for the studied population) as the reference (1 mg/dL = 0.0259 mmol per liter).

Since age is a major predictor for dementia in sub-group analysis, we examined the association between HDL-C and incident dementia according to age categories (binned according to the median age of ASPREE participants; <75 and ≥75 years).

Sensitivity analyses were conducted using the Fine–Gray competing risk model, which estimates the subdistribution HR for the association of levels of HDL-C and dementia with death considered as the competing risk. The analyses were further stratified by sex and frailty status. Analyses examining the associations between plasma HDL-C and incident dementia were repeated for Australian participants, participants who walked outside <30 min/day (as a measure of physical activity), and for those who had a stable BMI (within 5% change) in the first 2 years. Finally, stratified analyses were conducted according to *APOE* genotype to explore if effect of HDL-C is similar for incident dementia risk irrespective of *APOE* genetic variants (different *APOE* genotype categories included e1/e3:e2/e4, e2/e2, e2/e3, e3/e3, e3/e4, e4/e4).

Schoenfeld residual plots were used to evaluate the proportional hazards assumption, and Schoenfeld-type residuals assessed the proportional subdistribution hazard assumption of the Fine–Gray models. No evidence of violation of the underlying hazard assumptions was found in any analysis. Statistical significance was defined as a 2-sided P value < 0.05. Stata MP version 17 (StataCorp LP) was used for analysis. Data analysis was performed from October 2022 to January 2023.

### Ethics statements

The ASPREE clinical trial is registered with the International Standard Randomized Controlled Trial Number Register (ISRCTN83772183) and clinicaltrials.gov (NCT01038583). The ASPREE clinical trial was conducted following the World Medical Association Declaration of Helsinki 1964 as revised in 2008, the NHMRC Guidelines on Human Experimentation, the Federal Patient Privacy (HIPAA) law and the International Conference of Harmonisation Guidelines for Good Clinical Practice (ICH-GCP) guidelines and followed the Code of Federal Regulations. It was approved by the Monash University Human Research Ethics Committee (MUHREC) (IRB00002519; ethics #2006/745MC) and other allied institution ethics committees. The ASPREE Steering Committee was responsible for the trial's overall management and conduct.

### Role of the funding source

ASPREE was funded by National Institutes of Health, USA; and National Health and Medical Research Council in Australia; Monash University (Melbourne, VIC, Australia); and the Victorian Cancer Agency (Australia). The funders had no role in the design and conduct of this study; collection, management, analysis, and interpretation of the data and decision to submit the manuscript for publication. The content is solely the responsibility of the authors and does not necessarily represent the official views of the National Institutes of Health.

## Results

### Baseline characteristics

Among the 18,668 participants with plasma HDL-C measured at baseline, 850 (4.6%) cases of incident dementia were recorded over an average of 6.3 (SD 1.8) years of follow-up. On average, participants with an HDL-C level >80 mg/dL had a more favourable cardiovascular risk profile with lower frequencies of smoking, hypertension, diabetes, chronic renal disease and physical inactivity compared to the other categories (Table 1). However, HDL-C levels did not vary significantly between *APOE* genotype groups (Table 1).

**Table 1:** Baseline characteristics of participants according to HDL-C categories in the Aspirin in Reducing Events in the Elderly (ASPREE) cohort.

	≤40 mg/dL	40–60 mg/dL	60–80 mg/dL	>80 mg/dL
Age, mean (SD), y	74.7 (4.3)	75.0 (4.5)	75.2 (4.6)	75.5 (4.6)
Age in categories				
<75 years	1094 (61.8)	4818 (59.6)	3524 (57.8)	1492 (55.3)
≥75 years	676 (38.2)	3273 (40.5)	2574 (42.2)	1204 (44.7)
Female, n (%)	392 (22.2)	3732 (46.1)	4185 (68.6)	2222 (82.4)
Country of enrolment				
Australia	1529 (86.4)	7005 (86.6)	5332 (87.4)	2386 (88.5)
USA	241 (13.6)	1086 (13.4)	766 (12.6)	310 (11.5)
Low activity (walked outside <30 min), n (%)	775 (43.8)	3238 (40.0)	2263 (37.1)	916 (34.0)
BMI (kg/m^2^)	29.8 (4.3)	28.9 (4.6)	27.4 (4.5)	25.9 (4.6)
Current/former smoking, n (%)	893 (50.5)	3711 (45.9)	2562 (42.0)	1157 (42.9)
Current alcohol use, n (%)	1240 (70.1)	6028 (74.5)	4790 (78.6)	2221 (82.4)
Education >12 years of schooling, n (%)	671 (37.9)	3420 (42.3)	2637 (43.2)	1244 (46.2)
Hypertension, (%)	1432 (80.9)	6125 (75.7)	4407 (72.3)	1882 (69.8)
Chronic kidney disease (eGFR <60 ml/min), (%)	445 (25.8)	1544 (19.5)	949 (15.9)	404 (15.3)
Diabetes, n (%)	399 (22.5)	1040 (12.9)	410 (6.7)	139 (5.2)
Prefrail/Frail, (%)	783 (44.2)	3365 (41.6)	2434 (39.9)	1092 (40.5)
Incident dementia, n (%)	74 (4.2)	353 (4.4)	284 (4.6)	138 (5.1)
Total Cholesterol (mg/dl), (mean, SD)	180.4 (38.6)	196.2 (36.7)	209.4 (36.0)	222.0 (33.7)
Non-HDL-C (mg/dl), (mean, SD)	144.7 (38.3)	145.4 (36.3)	141.1 (36.1)	129.7 (33.4)
HDL-C PRS, mean (SD), z score (n = 13,349)	−0.61 (0.95)	−0.18 (0.95)	0.17 (0.95)	0.53 (0.94)
Apolipoprotein *E* genetic variants (n = 14,573)				
e1/e3:e2/e4 (n = 509)^a^	48 (9.5)	207 (41.1)	165 (32.7)	84 (16.8)
e2/e2 (n = 78)	2 (2.6)	32 (41.6)	30 (39.0)	13 (16.9)
e2/e3 (n = 1964)	168 (8.8)	789 (41.4)	630 (33.1)	317 (16.7)
e3/e3 (n = 8775)	801 (9.4)	3679 (43.0)	2871 (33.6)	1202 (14.1)
e3/e4 (n = 3020)	295 (10.0)	1319 (44.8)	928 (31.5)	403 (13.7)
e4/e4 (n = 227)	38 (17.0)	95 (42.6)	61 (27.4)	29 (13.0)

a ambiguous genotypes: since e1/e3 and e2/e4 have the same genotypes for the two SNPs, they were combined together as one group.

Participants diagnosed with dementia were more likely to be older, female, less physically active, less likely to be a current or former smoker or to report alcohol use, but with a higher prevalence of prefrailty/frailty at baseline (Supplementary Table S1). The prevalence of hypertension, diabetes, chronic kidney disease was similar irrespective of incident dementia diagnosis status, although, *APOE* e3/e4 and *APOE* e4/e4 genotype groups were more prevalent in participants with incident dementia (Supplementary Table S1).

### HDL-C levels and incident dementia

The associations between HDL-C levels and incident dementia are presented in Table 2. In the group with very high HDL-C levels (>80 mg/dL) the rate of incident dementia was 81 events per 10,000 person-years compared to 69 events per 10,000 person-years amongst those with an HDL-C of 40–60 mg/dL. After adjustment for age, country of enrolment, prefrailty/frailty status, physical activity, alcohol use, smoking status, level of education, aspirin allocation, non HDL-C level, baseline cognitive function, hypertension, diabetes, chronic kidney disease, participants who had very high HDL-C (>80 mg/dL) had a 27% higher risk of incident dementia (HR 1.27, 95% CI 1.03, 1.58), compared with the reference category (HDL-C 40–60 mg/dL) (model 4). When *APOE* genotype was added to the model, the effect of high HDL-C on dementia risk remained largely unchanged (HR 1.29, 95% CI 1.00, 1.67).

**Table 2:** Association between baseline high-density plasma cholesterol (HDL-C) levels and incident dementia (HR, 95% CI).

	HDL levels			
	<40 mg/dl	40–60 mg/dl	60–80 mg/dl	>80 mg dl
Total population				
No. of participants	1770	8091	6098	2709
Incident dementia (n, %)	74 (4.2)	353 (4.4)	284 (4.7)	139 (5.1)
Rate/10,000 person-year (95% CI)	67 (53–83)	69 (62–77)	73 (65–82)	81 (68–96)
Model 1	0.95 (0.74–1.23)	1	1.09 (0.93–1.28)	1.21 (0.99–1.49)
Model 2	0.93 (0.72–1.20)	1	1.10 (0.94–1.29)	1.22 (1.00–1.50)
Model 3	0.86 (0.67–1.11)	1	1.18 (1.00–1.38)	1.29 (1.05–1.58)
Model 4	0.88 (0.68–1.14)	1	1.17 (0.99–1.38)	1.27 (1.03–1.58)
Model 5^a^	0.86 (0.63–1.16)	1	1.16 (0.96–1.41)	1.29 (1.00–1.67)
Model 6^b^	0.85 (0.62–1.17)	1	1.18 (0.96–1.45)	1.31 (0.99–1.73)
<75 years of age				
No. of participants	1094	4818	3524	1500
Incident dementia (n, %)	24 (2.2)	129 (2.7)	95 (2.7)	38 (2.5)
Rate/10,000 person-year (95% CI)	34 (23–51)	42 (35–50)	42 (34–51)	39 (28–53)
Model 1	0.79 (0.51–1.22)	1	1.03 (0.78–1.36)	0.98 (0.68–1.43)
Model 2	0.76 (0.49–1.19)	1	1.05 (0.80–1.38)	1.01 (0.69–1.47)
Model 3	0.75 (0.48–1.17)	1	1.07 (0.82–1.41)	1.06 (0.73–1.55)
Model 4	0.76 (0.49–1.19)	1	1.05 (0.79–1.39)	1.02 (0.68–1.51)
Model 5^c^	0.70 (0.42–1.15)	1	1.05 (0.76–1.45)	1.08 (0.69–1.70)
Model 6^d^	0.71 (0.42–1.20)	1	1.05 (0.75–1.47)	1.05 (0.63–1.71)
>75 years of age				
No. of participants	676	3273	2574	1209
Incident dementia (n, %)	50 (7.4)	224 (6.8)	189 (7.3)	101 (8.4)
Rate/10,000 person-year (95% CI)	121 (92–160)	111 (97–127)	118 (103–137)	137 (112–166)
Model 1	1.06 (0.77–1.44)	1	1.12 (0.92–1.36)	1.33 (1.04–1.70)
Model 2	1.04 (0.76–1.42)	1	1.13 (0.93–1.38)	1.33 (1.04–1.70)
Model 3	1.00 (0.74–1.37)	1	1.15 (0.94–1.40)	1.39 (1.08–1.77)
Model 4	1.02 (0.74–1.39)	1	1.17 (0.96–1.43)	1.42 (1.10–1.83)
Model 5^e^	0.93 (0.64–1.36)	1	1.20 (0.94–1.52)	1.41 (1.04–1.91)
Model 6^f^	0.93 (0.62–1.37)	1	1.23 (0.95–1.59)	1.49 (1.06–2.08)

Model 1: adjusted for age, sex.

Model 2: adjusted for age, sex, frailty.

Model 3: adjusted for age, sex, frailty, country of enrolment, physical activity, alcohol use, smoking status, level of education, 100 mg Aspirin, baseline 3MS overall score.

Model 4: adjusted for age, sex, frailty, country of enrolment, physical activity, alcohol use, smoking status, level of education, 100 mg Aspirin, nonHDL-C, hypertension, diabetes, chronic kidney disease, baseline 3MS overall score.

Model 5: adjusted for age, sex, frailty, country of enrolment, physical activity, alcohol use, smoking status, level of education, 100 mg Aspirin, baseline 3MS overall score, nonHDL-C, hypertension, diabetes, chronic kidney disease, APOE.

Model 6: adjusted for age, sex, frailty, country of enrolment, physical activity, alcohol use, smoking status, level of education, 100 mg Aspirin, baseline 3MS overall score, nonHDL-C, hypertension, diabetes, chronic kidney disease, HDL-C genetic PRS and 10 principal components of population structure.

a n = 13,903.

b n = 12,690.

c n = 8535.

d n = 7856.

e n = 5864.

f n = 5297.

### Age, HDL-C levels and incident dementia

**Fig. 1:**
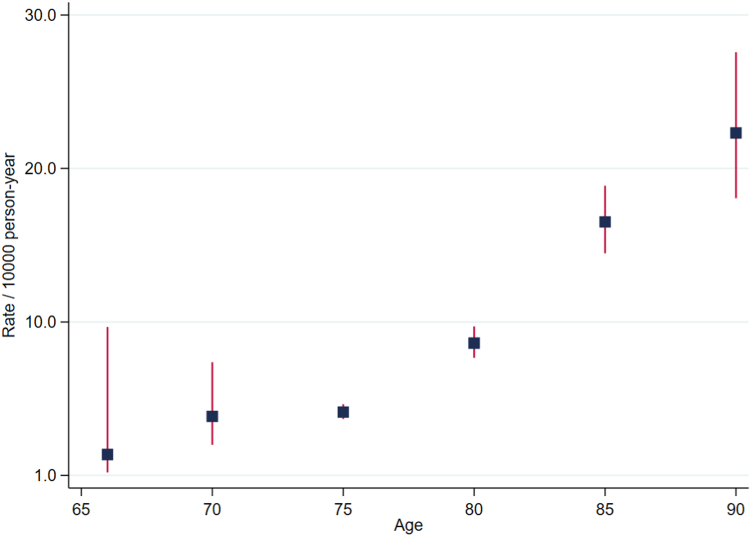
**Incidence rates of dementia for age categories spanning five years each**.

The incidence rates of dementia were higher in participants older than 75 years of age compared with the participants younger than 75 years of age. The incidence rates were 11.8/1000 person-year and 4.1/1000 person-year respectively (P < 0.0001). Fig. 1 shows the incidence rates of dementia for age categories spanning five years each. Participants who were older than 75 years of age had on average, higher levels of HDL-C compared to participants younger than 75 years, and there was a higher percentage of participants 75 years or older who developed incident dementia compared to those younger than 75 (7.3% vs 2.6%) (Table 2, Supplementary Table S2, and Supplementary Figure S1).

The rate of incident dementia among the participants younger than 75 was 39 events per 10,000 person-years in those with HDL-C levels >80 mg/dL compared with 42 events per 10,000 person-years in the reference group (HDL-C 40–60 mg/dL). However, for participants 75 years and older, the rate of incident dementia was markedly higher in the high HDL-C group (>80 mg/dL) (111 events per 10,000 person-years vs 137 events per 10,000 person-years compared with the reference group). This equated to a statistically significant 42% increase in dementia risk in participants older than 75 years in the fully adjusted model (HR 1.42, 95% CI 1.10, 1.83), which took account of sex, country of enrolment, prefrailty/frailty status, physical activity, alcohol use, smoking status, level of education, aspirin allocation, nonHDL-C, baseline cognitive function, hypertension, diabetes, chronic kidney disease (model 4). Adding *APOE* genetic variants to the model did not alter the risk (HR 1.41, 95% CI 1.04, 1.91). No similar association was observed for the participants younger than 75 years of age.

### HDL-C PRS

Amongst the 13,007 participants where genetic data was available, the HDL-C-PRS (not including *APOE*) was added to the fully adjusted model to determine whether genetic influences on HDL-C levels could explain the association observed between elevated HDL-C and dementia risk. After this adjustment the association between incident dementia and high HDL-C (>80 mg/dL) remained statistically significant for the overall population, and for the older subgroup of participants aged ≥75 years (Table 2, *model 6*).

### Consideration of nonlinearity

**Fig. 2:**
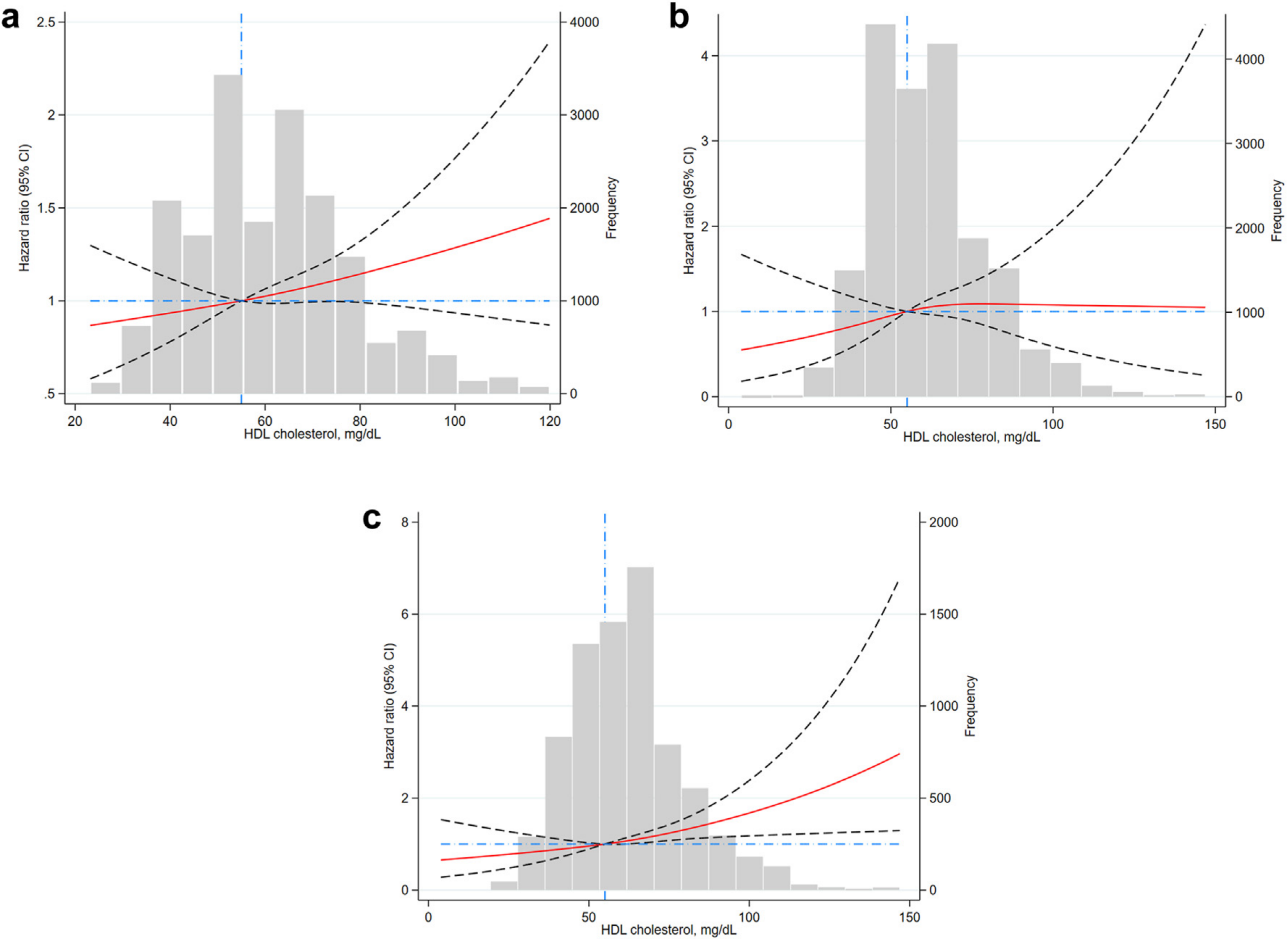
**Spline curve showing the association Between High-Density Lipoprotein (HDL) Cholesterol Levels and Dementia:** a. Total population. b. younger than 75 years of age. c. older than 75 years of age.

Using restricted cubic spline curves adjusted for the previously mentioned covariates (but not including *APOE,* and HDL-C PRS) a near–linear association between HDL-C levels and dementia risk was demonstrated for all participants, and participants 75 years and older, reflecting an increasing risk of incident dementia as levels of HDL-C increase (Fig. 2).

### Sensitivity analyses

Results remained similar in the competing risk analyses (Supplementary Table S3) and after the analyses were stratified by sex and prefrailty/frailty status (Supplementary Table S4). They were also unchanged after excluding those who were highly physically active, those whose weight remained stable within 2 years of recruitment and with the inclusion of only Australian participants (Supplementary Table S5). Amongst those with *APOE* genetic categories (e3/e4, and e4/e4) linked to dementia the results were unchanged (Supplementary Table S6).

## Discussion

The principal finding of this study is that very high levels of plasma HDL-C (>80 mg/dL) are associated with an increased risk of incident dementia in initially-healthy older people. This association was statistically significant in people aged 75 years and older. The increased dementia risk associated with high HDL-C levels appeared to be independent of traditional dementia risk factors, including physical activity level, alcohol intake, education, diabetes or smoking.

The associations also remained significant after adjusting for genetic influences on HDL-C levels through both *APOE* genotype and an HDL-C-PRS. In addition, HDL-C levels were little different in those with the ‘high dementia risk’ *APOE* genotypes (e.g. e3/e4 and e4/e4) compared with the reference *APOE* genotypes (e.g. e3/e3) further demonstrating that the increased dementia risk conferred by a very high HDL-C was unlikely to be confounded through this association.

Both genetic and lifestyle factors strongly influence HDL-C levels, meaning a purely genetic explanation is not necessarily expected. Previous Mendelian randomization studies have not yet supported a causal role for HDL-C genetic variability in the development of dementia.^6^^,^^18^ Also, studies in which HDL-C levels have been increased by the inhibition of cholesteryl ester transfer protein (CETP) do not appear to have influenced dementia risk.^19^ Although ASPREE is a randomized controlled trial of 100 mg enteric coated aspirin, these results are not due to the effect of aspirin on dementia, as our previous analyses showed that aspirin did not affect the incidence of dementia.^12^

Our findings contrast with those of a recently published meta-analysis of data from 100 studies suggesting that HDL-C levels were unrelated to dementia.^20^ Most of the studies included in this meta-analysis had categorised HDL-C in tertiles or quartiles without specifically identifying individuals with very high HDL-C levels.^6^ However, our results are similar to recently published data from the Copenhagen General Population Study and the Copenhagen City Heart Study that suggested a relationship between very high HDL-C and dementia in a relatively younger population with prevalent comorbidities.^6^ In these studies, the observed relationship was restricted to very high HDL-C levels (as noted in our study) and the association was also observed to be independent of *APOE* genotype. Our results extend the Copenhagen studies by including older participants who were initially healthy and cognitively intact.

The explanation as to why high HDL-C might be associated with an increased risk of dementia is unclear. Earlier studies linking HDL-C to cognitive function had suggested that moderately high HDL levels were associated with better cognitive function and that low HDL-C levels were associated with an increased severity of Alzheimer's disease. However, HDLs are complex particles with various physiological functions likely determined by proteins and other compounds carried in their phospholipid coating.^21^ Plasma HDL-C levels do not necessarily reflect functional aspects of lipid transport and at very high levels this may be dysfunctional.^22^ The possibility also exists that increased dementia and very high HDL are both consequences of a separate and unrelated pathology.

With the challenge of aging populations in most developed countries, dementia is becoming an increasing social and economic issue. Currently, there is a lack of proven non-invasive biomarkers of early dementia risk and no well-established interventions to slow cognitive decline. Circulating HDL-C levels are easily measurable, potentially modifiable, and therefore may become a useful dementia biomarker, albeit relevant to only a small fraction of individuals. Further analysis of the role of high HDL-C may also provide new insights into the pathogenesis of dementia and may help explain other unfavourable impacts of high HDL-C such as higher rates of mortality,^1^^,^^2^^,^^23^ adverse cardiovascular outcomes,^2^^,^^16^ and fractures.^5^ Recent intervention studies suggesting a role for certain monoclonal antibody derived therapies such as donanemab may provide a stronger rationale for the identification and treatment of individuals found to be at high risk of dementia though high risk *APOE* genotypes or a very high HDL level.^24^

### Strength and limitations

Strengths of this study include its focus on healthy community-dwelling individuals aged ≥70 years who were cognitively healthy where dementia is a major concern of loss of independence. The study involved intensive and rigorous data collection, and expert adjudication of dementia as a primary trial endpoint. Despite the usual constraints of an observational study, the internal consistency of the findings amongst key subgroups provides support for their validity.

On the other hand, the results might be generalizable only to healthy Caucasian adults. Heavy alcohol intake (which is a recognised cause of high HDL-C) may not have been adequately captured via self-report and may have resulted in a degree of unadjusted confounding. The HDL-C-PRS (based only on common variants identified by GWAS) does not capture all genetic variation or heritability that contributes to plasma HDL-C levels, including rare variants, and therefore does not fully exclude a genetic contribution to our findings. The possibility that dysfunctional HDL particles could explain the observed results (rather than HDL-C levels themselves) also requires further evaluation.

### Conclusions

Overall the results provide evidence that very high HDL-C levels are associated with incident dementia risk in both older males and females, and that this association appears to be independent of traditional dementia risk factors, and genetic factors. Given dementia is the third major cause of disability worldwide,^25^ these findings are timely and may suggest that identifying individuals with very high HDL-C could act as a new strategy for the early identification of high-risk individuals. However, further research is needed to determine the pathophysiological explanation for these findings.

## Contributors

SMH and JMCN conceived and designed the study. AMT, PL; TC, LJB, JR, MEE, and JMCN contributed to the data collection. SMH, and JMCN generated the data for analyses. SMH did the statistical analysis with guidance from AMT, and JMCN. SMH and JMCN verified the underlying data. SMH, CR, AMT, PL, LJB, CY, GFW, MEE, ZZ, JTN, and JMCN were responsible for data interpretation and validation. SMH, and JMCN drafted the manuscript. All the authors critically revised the manuscript for important intellectual content, and approved the final manuscript. SMH and JMCN had full access to all of the data in the study. SMH, and JMCN were responsible for the decision to submit the manuscript. All authors had final responsibility for the decision to submit for publication.

## Data sharing statement

The datasets generated during the current study are available from the corresponding author with permission of the ASPREE principal investigators.

## Declaration of interests

SMH is the recipient of National Health and Medical Research Council (NHMRC) Early Career Fellowship (APP1142198), JMCN is supported through an NHMRC Leadership Fellowship (IG 1173690). PL is supported by a National Heart Foundation Future Leader Fellowship (102604). JR is supported by an NHMRC Leadership Investigator Grant (2016438). TC is supported by an Australian Research Council Future Fellowship (FT220100294), and has received honoraria from Roche for lectures. GFW received grants from Arrowhead Pharma, Amgen, Novartis, Silence Therapeutics; consulting fee from Amgen, Novartis, Sanofi, Esperion; honoraria from Amgen, Novartis, Sanofi; support for attending meetings from Novartis, Silence Therapeutics. No other disclosures are reported by the other authors.
