# Supplementary material for: Association of plasma high-density lipoprotein cholesterol level with risk of incident dementia: a cohort study of healthy older adults
Source: Lancet Reg Health West Pac. 2023 Nov 29;43:100963. doi: 10.1016/j.lanwpc.2023.100963 (PMC10920036; doi:10.1016/j.lanwpc.2023.100963)
Supplement: Supplementary Tables S1–S6 and Supplementary Figure S1 [file mmc1.pdf]

Supplemental Online Content

Association of Plasma High-Density Lipoprotein Cholesterol Level with Risk of Incident Dementia: A Cohort Study of Healthy Older Adults.

Supplementary Table S1: Baseline characteristics of participants: Overall, and dementia status in the Aspirin in Reducing Events in the Elderly (ASPREE) cohort

Supplementary Table S2: Rate of incident dementia and plasma high-density lipoprotein cholesterol levels according to age category

Supplementary Table S3: Association between baseline high-density plasma cholesterol (HDL-C) levels and incident dementia (HR, 95% CI) Fine-Gray competing risk analyses

Supplementary Table S4: Association between baseline plasma high-density lipoprotein cholesterol levels and incident dementia stratified by sex, and prefrailty/frailty status (HR, 95% CI)

Supplementary Table S5: Association between baseline plasma high-density lipoprotein cholesterol levels and incident dementia: analyses restricted to Australians, participants who did not report high physical activity and participants who had a stable weight withing two years of recruitment (HR, 95% CI)

Supplementary Table S6: Association between baseline plasma high-density lipoprotein cholesterol levels and incident dementia according to Apolipoprotein *E* genetic variants among participants >75 years of age (HR, 95% CI)

Supplementary Figure S1: Distribution of plasma high-density lipoprotein cholesterol: a. Males <75 years of age b. Males ≥75 years of age c. Females <75 years of age d. Females ≥75 years of age

Supplementary Table S1: Baseline characteristics of participants: Overall, and dementia status in the Aspirin in Reducing Events in the Elderly (ASPREE) cohort

	Overall	No dementia	Incident dementia	P value
N (%)	18,668 (100)	17818 (95·5)	850 (4·6)	
Age, mean (SD), y	75·1 (4·5)	75·0 (4·5)	77·9 (5·1)	<0·0001
Female, n (%)	10,782 (56·4)	10,320 (56·6)	462 (53·0)	0·04
Country of enrolment				0·47
Australia	16,703 (87·4)	15,934 (87·4)	769 (88·2)	
USA	2411 (12·6)	2,308 (12·7)	103 (11·8)	
Low activity (walked outside <30 minutes), n (%)	7,366 (38·5)	6991 (38·3)	375 (43·0)	0·01
BMI (kg/m2)	28·1 (4·7)	28·1 (4·7)	27·1 (4·7)	0·001
Current/former smoking, n (%)	8,534 (44·7)	8,171 (44·8)	363 (41·6)	0·07
Current alcohol use, n (%)	14,642 (76·6)	14009 (76·8)	633 (72·6)	0·004
Education, n (%)				0·08
≤12 years of schooling	10955 (57·3)	10,430 (57·2)	525 (60·2)	
>12 years of schooling	8158 (43·7)	7811 (42·8)	347 (39·8)	
Hypertension, (%)	14,195 (74·3)	13,544 (74·3)	651 (74·7)	0·79
Chronic kidney disease (eGFR <60ml/min), (%)	3,427 (18·4)	3,246 (18·2)	181 (21·1)	
Diabetes, n (%)	2,045 (10·7)	1,943 (10·7)	102 (11·7)	0·32
Prefrail/Frail, (%)	7,868 (41·2)	7,375 (40·4)	493 (56·5)	<0·0001
On trial medication, (%)	9525 (49·8)	9097 (49·9)	428 (49·1)	0·65
3MS overall score, mean (SD)	93·4 (4·6)	93·6 (4·5)	89·5 (5·7)	<0·0001
HDL-C PRS, mean (SD), z score (n=13,349)	0·0 (1)	0·003 (1·00)	-0·85 (0·92)	0·04
APOE (n=14573)				<0·0001
e1/e3:e2/e4	509 (3·5)	487 (3·5)	22 (3·6)	
e2/e2	78 (0·5)	77 (0·6)	1 (0·2)	
e2/e3	1964 (13·5)	1908 (13·7)	56 (9·2)	
e3/e3	8775 (60·2)	8492 (60·8)	283 (46·2)	
e3/e4	3020 (20·7)	2798 (20·0)	222 (36·3)	
e4/e4	227 (1·6)	199 (1·4)	28 (4·6)	
HDL Cholesterol (mg/dl), (mean, SD)	61·2 (17·7)	61·2 (17·7)	62·0 (18·1)	
HDL Cholesterol category, n (%)				
≤40 mg/dL	1770 (9·5)	1696 (9·5)	74 (8·7)	
40-60 mg/dL	8091 (43·3)	7738 (43·4)	353 (41·5)	
60-80 mg/dL	6098 (32·7)	5814 (32·6)	284 (33·4)	
>80 mg/dL	2709 (14·5)	2570 (14·4)	139 (16·4)	
Total Cholesterol (mg/dl), (mean, SD)	202·7 (38·1)	202·7 (38·0)	202·2 (40·4)	0·67
Non-HDL-C (mg/dl), (mean, SD)	141·6 (36·5)	141·7 (36·4)	140·5 (38·3)	0·37

Supplementary Table S2: Rate of incident dementia and plasma high-density lipoprotein cholesterol levels according to age category

	<75 years	≥75 years	p-value
Incident dementia, n (%)	294 (2·6)	578 (7·3)	<0·001
HDL Cholesterol (mg/dl), (mean, SD)	60·7 (17·7)	61·9 (17·8)	<0·001
HDL Cholesterol category, n (%)			<0·001
≤40 mg/dL	1094 (10·0)	676 (8·7)	
40-60 mg/dL	4818 (44·1)	3273 (42·3)	
60-80 mg/dL	3524 (32·2)	2574 (33·3)	
>80 mg/dL	1500 (13·7)	1209 (15·6)	
Total Cholesterol (mg/dl), (mean, SD)	203·5 (38·2)	201·5 (37·9)	0·001
Non-HDL-C (mg/dl), (mean, SD)	142·9 (36·4)	139·7 (36·4)	<0·001

Supplementary Table S3: Association between baseline high-density plasma cholesterol (HDL-C) levels and incident dementia (HR, 95% CI) Fine-Gray competing risk analyses

	HDL levels			
	<40 mg/dl	40-60 mg/dl	60-80 mg/dl	>80mg dl
**Total population**				
N (%)	74 (4·2)	353 (4·4)	284 (4·7)	139 (5·1)
Rate /10,000 person-year (95% CI)	67 (53-84)	69 (62-77)	73 (65-82)	81 (69-96)
Model 1	0·95 (0·74-1·23)	1	1·09 (0·93-1·28)	1·21 (0·99-1·49)
Model 2	0·93 (0·73-1·20)	1	1·10 (0·94-1·29)	1·22 (1·00-1·50)
Model 3	0·91 (0·70-1·17)	1	1·12 (0·96-1·31)	1·27 (1·04-1·56)
Model 4	0·92 (0·71-1·19)	1	1·12 (0·95-1·32)	1·27 (1·03-1·58)
Model 5[a]	0·86 (0·63-1·16)	1	1·16 (0·96-1·42)	1·29 (1·00-1·67)
Model 6[b]	0·85 (0·62-1·17)	1	1·18 (0·96-1·45)	1·31 (0·99-1·73)
**<75 years of age**				
N (%)	24 (2·2)	129 (2·7)	95 (2·7)	38 (2·5)
Rate /10,000 person-year (95% CI)	34 (23-51)	42 (35-50)	42 (34-51)	39 (28-53)
Model 1	0·79 (0·51-1·22)	1	1·03 (0·78-1·35)	0·98 (0·68-1·43)
Model 2	0·76 (0·49-1·19)	1	1·05 (0·80-1·38)	1·01 (0·69-1·47)
Model 3	0·75 (0·49-1·17)	1	1·08 (0·82-1·41)	1·06 (0·73-1·55)
Model 4	0·76 (0·49-1·19)	1	1·05 (0·79-1·39)	1·02 (0·68-1·51)
Model 5[c]	0·70 (0·42-1·15)	1	1·05 (0·76-1·45)	1·08 (0·69-1·70)
Model 6[d]	0·71 (0·42-1·20)	1	1·05 (0·75-1·48)	1·05 (0·64-1·71)
**>75 years of age**				
N (%)	50 (7·4)	224 (6·8)	189 (7·3)	101 (8·4)
Rate /10,000 person-year (95% CI)	121 (92-160)	111 (97-127)	118 (103-137)	136 (112-166)
Model 1	1·06 (0·77-1·44)	1	1·12 (0·92-1·37)	1·33 (1·04-1·70)
Model 2	1·04 (0·76-1·42)	1	1·13 (0·93-1·38)	1·33 (1·04-1·70)
Model 3	1·00 (0·74-1·37)	1	1·15 (0·94-1·40)	1·39 (1·08-1·77)
Model 4	1·02 (0·74-1·39)	1	1·17 (0·96-1·43)	1·42 (1·10-1·83)
Model 5[e]	0·93 (0·64-1·36)	1	1·20 (0·94-1·52)	1·41 (1·04-1·91)
Model 6[f]	0·93 (0·62-1·37)	1	1·23 (0·95-1·59)	1·49 (1·07-2·08)

Model 1: adjusted for age, sex

Model 2: adjusted for age, sex, frailty

Model 3: adjusted for age, sex, frailty, country of enrolment, physical activity, alcohol use, smoking status, level of education, 100mg Aspirin, baseline 3MS overall score

Model 4: adjusted for age, country of enrolment, frailty, physical activity, alcohol use, smoking status, level of education, 100mg Aspirin, nonHDL-C, hypertension, diabetes, chronic kidney disease, baseline 3MS overall score

Model 5: adjusted for age, country of enrolment, frailty, physical activity, alcohol use, smoking status, level of education, 100mg Aspirin, baseline 3MS overall score, nonHDL-C, hypertension, diabetes, chronic kidney disease, APOE

Model 6: adjusted for age, country of enrolment, frailty, physical activity, alcohol use, smoking status, level of education, 100mg Aspirin, baseline 3MS overall score, nonHDL-C, hypertension, diabetes, chronic kidney disease, HDL-C genetic PRS and 10 principal components of population structure

Supplementary Table S4: Association between baseline plasma high-density lipoprotein cholesterol levels and incident dementia stratified by sex, and prefrailty/frailty status (HR, 95% CI)

	HDL levels			
	<40 mg/dl	40-60 mg/dl	60-80 mg/dl	>80mg dl
**Stratified by sex[a]**				
Male				
All	0·83 (0·57-1·19)	1	1·13 (0·97-1·72)	1·16 (0·71-1·94)
<75 years	0·63 (0·34-1·17)	1	1·14 (0·72-1·79)	1·04 (0·47-2·28)
≥75 years	0·95 (0·61-1·49)	1	1·26 (0·88-1·80)	1·28 (0·97-2·29)
Female				
All	1·04 (0·55-1·96)	1	0·99 (0·76-1·32)	1·21 (0·86-1·70)
<75 years	1·11 (0·46-2·65)	1	0·80 (0·50-1·27)	0·89 (0·50-1·58)
≥75 years	0·85 (0·37-2·00)	1	1·05 (0·87-1·49)	1·35 (0·99-2·03)
**Stratified by Prefrailty/Frailty status[b]**				
Not prefrail/frail				
All	0·90 (0·55-1·40)	1	1·12 (0·83-1·51)	1·45 (0·98-2·14)
<75 years	0·69 (0·35-1·36)	1	1·20 (0·79-1·83)	1·13 (0·61-2·09)
≥75 years	1·01 (0·56-1·84)	1	0·98 (0·66-1·46)	1·63 (1·01-2·63)
Prefrail/frail				
All	0·81 (0·53-1·25)	1	1·18 (0·90-1·55)	1·19 (0·80-1·76)
<75 years	0·66 (0·31-1·39)	1	0·76 (0·44-1·28)	0·96 (0·48-1·94)
≥75 years	0·87 (0·50-1·38)	1	1·32 (0·84-1·83)	1·31 (0·96-2·06)

Adjusted for age, country of enrolment, frailty, physical activity, alcohol use, smoking status, levels of education, 100mg Aspirin, nonHDL-C, hypertension, diabetes, chronic kidney disease
[a]sex was not adjusted [b]prefrailty/frailty was not adjusted

Supplementary Table S5: Association between baseline plasma high-density lipoprotein cholesterol levels and incident dementia: analyses restricted to Australians, participants who did not report high physical activity and participants who had a stable weight withing two years of recruitment (HR, 95% CI)

	HDL levels			
	<40 mg/dl	40-60 mg/dl	60-80 mg/dl	>80mg dl
**Excluding the US population (n=15,895)[a]**				
All	0·86 (0·62-1·20)	1	1·21 (0·97-1·50)	1·25 (0·93-1·69)
<75 years	0·64 (0·37-1·11)	1	1·06 (0·75-1·50)	0·92 (0·54-1·57)
>75 years	1·00 (0·66-1·50)	1	1·27 (0·97-1·65)	1·48 (1·04-2·10)
**Excluding those with high physical activity (n=4,677)[b]**				
All	0·71 (0·43-1·17)	1	1·57 (1·15-2·14)	1·59 (1·15-2·51)
<75 years	0·70 (0·33-1·50)	1	1·11 (0·66-1·88)	1·23 (0·57-2·67)
>75 years	0·65 (0·35-1·23)	1	1·68 (1·16-2·43)	1·78 (1·06-3·00)
**Excluding those who did not have a stable weight within 2 years of recruitment (n=8924)**				
All	0·81 (0·53-1·21)	1	1·19 (0·92-1·55)	1·27 (0·90-1·82)
<75 years	0·74 (0·38-1·46)	1	0·96 (0·60-1·52)	0·92 (0·47-1·79)
>75 years	0·83 (0·50-1·39)	1	1·33 (0·97-1·82)	1·51 (1·00-2·31)

Adjusted for age, country of enrolment, frailty, physical activity, alcohol use, smoking status, levels of education, 100mg Aspirin, nonHDL-C, hypertension, diabetes, chronic kidney disease
[a] not adjusted for country of enrolment [b] not adjusted for physical activity

Supplementary Table S6: Association between baseline plasma high-density lipoprotein cholesterol levels and incident dementia according to Apolipoprotein *E* genetic variants among participants ≥75 years of age (HR, 95% CI)

	HDL levels			
	<40 mg/dl	40-60 mg/dl	60-80 mg/dl	>80mg dl
e1/e3:e2/e4 (n=196)	-	1	0·56 (0·02, 13·4)	8·50 (0·60, 12·0)
e2/e2 (n=33)	-	1	-	-
e2/e3 (n=801)	0·59 (0·16-2·18)	1	1·42 (0·66-3·05)	1·95 (0·72-5·32)
e3/e3 (n=3444)	1·24 (0·72-2·13)	1	1·49 (1·03-2·15)	1·81 (1·12-2·91)
e3/e4 (n=1029)	0·79 (0·38-1·66)	1	0·98 (0·62-1·54)	1·20 (0·95-1·93)
e4/e4 (n=61)	0·51 (0·01-39·9)	1	2·06 (0·07-64·2)	6·39 (0·84-282)

Adjusted for age, country of enrolment, frailty, physical activity, alcohol use, smoking status, level of education, 100mg Aspirin, baseline 3MS overall score, nonHDL-C, hypertension, diabetes, chronic kidney disease, HDL-C genetic PRS

Supplementary Figure S1: Distribution of plasma high-density lipoprotein cholesterol: a. Males <75 years of age b. Males ≥75 years of age c. Females <75 years of age d. Females ≥75 years of age

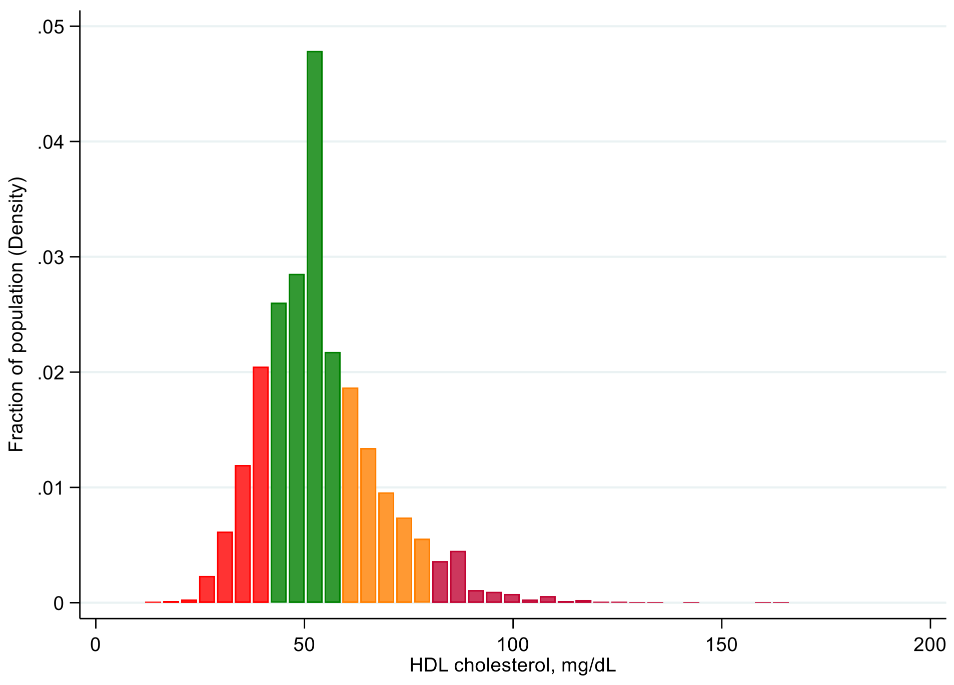

a

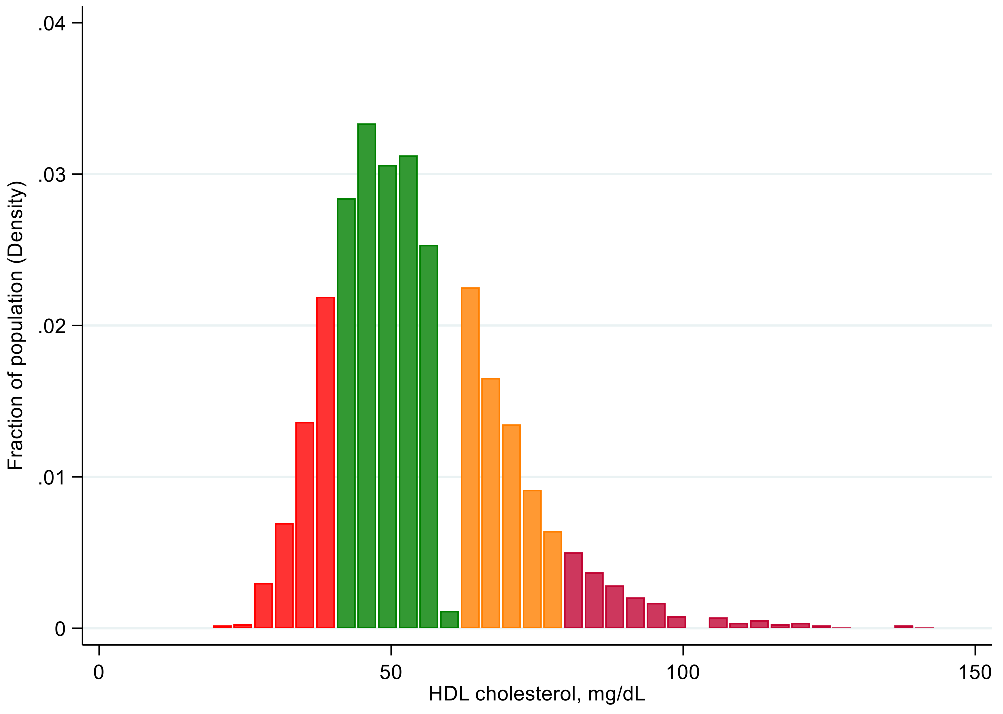

b

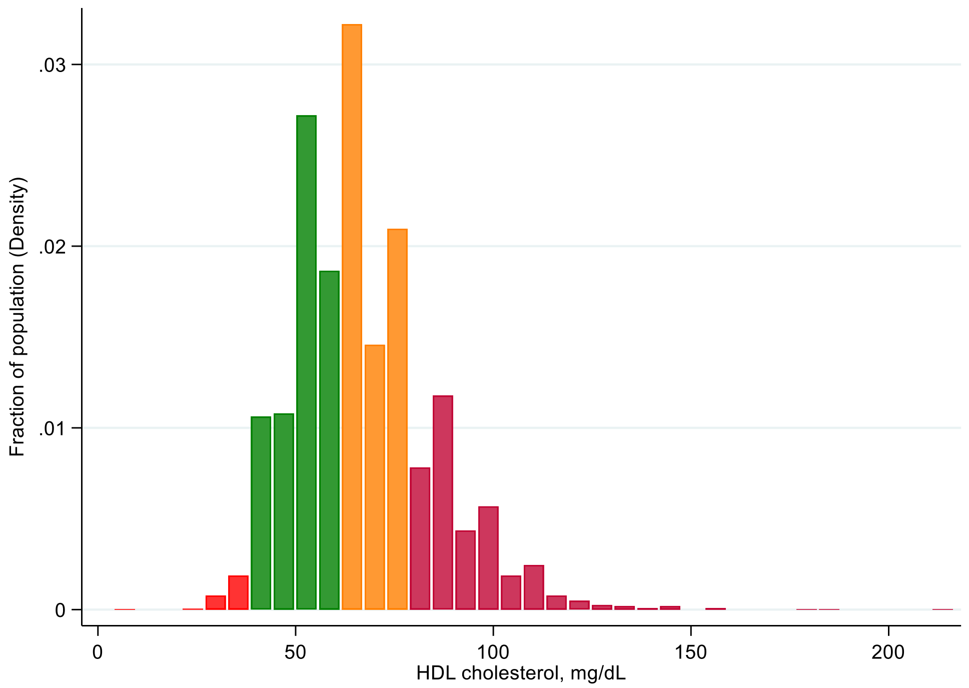
Fraction of population (Density)
.03
.02
.01
0
0
50
100
150
200
HDL cholesterol, mg/dL
c

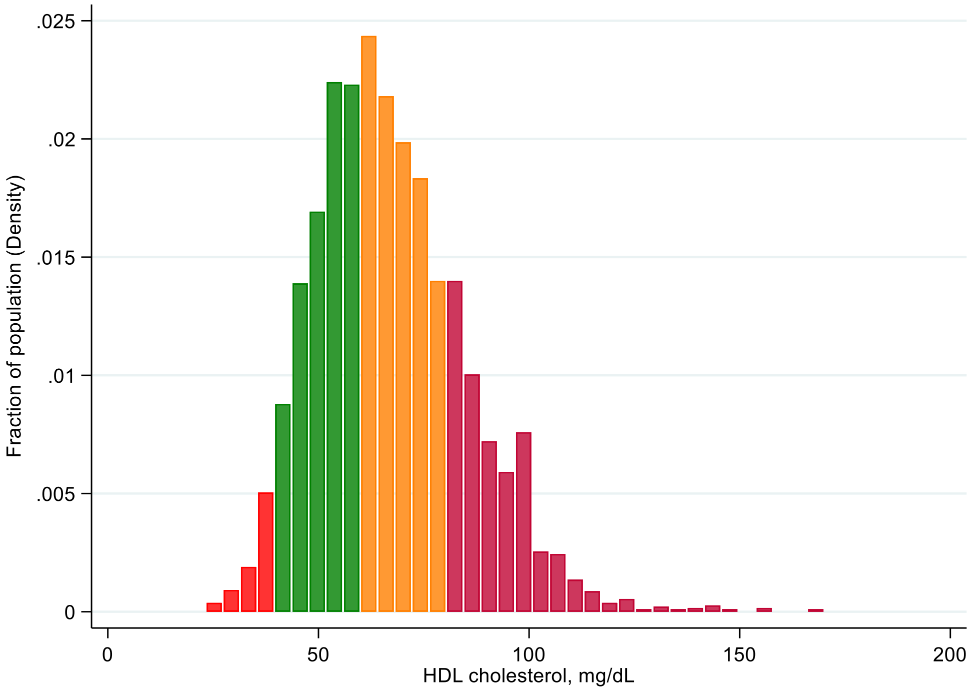
Fraction of population (Density)
.025
.02
.015
.01
.005
0
0
50
100
150
200
HDL cholesterol, mg/dL
d
